# Comparative and phylogenetic analysis of complete chloroplast genomes from five *Artemisia* species

**DOI:** 10.3389/fpls.2022.1049209

**Published:** 2022-11-21

**Authors:** Zhaohui Lan, Yuhua Shi, Qinggang Yin, Ranran Gao, Chunlian Liu, Wenting Wang, Xufang Tian, Jiawei Liu, Yiying Nong, Li Xiang, Lan Wu

**Affiliations:** ^1^ Key Laboratory of Beijing for Identification and Safety Evaluation of Chinese Medicine, Artemisinin Research Center, Institute of Chinese Materia Medica, China Academy of Chinese Medical Sciences, Beijing, China; ^2^ College of Pharmacy, Hubei University of Chinese Medicine, Wuhan, China; ^3^ Department of product development, Hubei Aiaitie Health Technology Co., LTD, Huanggang, China

**Keywords:** *Artemisia* Linn., chloroplast genome, genome comparison, species identification, phylogenetic analysis, simple sequence repeat

## Abstract

*Artemisia* Linn. is a large genus within the family Asteraceae that includes several important medicinal plants. Because of their similar morphology and chemical composition, traditional identification methods often fail to distinguish them. Therefore, developing an effective identification method for *Artemisia* species is an urgent requirement. In this study, we analyzed 15 chloroplast (cp) genomes, including 12 newly sequenced genomes, from 5 *Artemisia* species. The cp genomes from the five *Artemisia* species had a typical quadripartite structure and were highly conserved across species. They had varying lengths of 151,132–151,178 bp, and their gene content and codon preferences were similar. Mutation hotspot analysis identified four highly variable regions, which can potentially be used as molecular markers to identify *Artemisia* species. Phylogenetic analysis showed that the five *Artemisia* species investigated in this study were sister branches to each other, and individuals of each species formed a monophyletic clade. This study shows that the cp genome can provide distinguishing features to help identify closely related *Artemisia* species and has the potential to serve as a universal super barcode for plant identification.

## Introduction

*Artemisia* Linn. is a large genus within the family Asteraceae comprising commonly used herbs that have a long history of medicinal use (Watson et al., 2002; Riggins, 2008; Kim G. B. et al., 2020). Among the various compounds present in these plants, terpenoids represent the main effective component. Modern pharmacological studies have shown that *Artemisia* medicinal plants exert diuretic, expectorant, antiinflammatory, hemostatic, hypotensive, and antiallergic effects (Chen et al., 2021; Hsueh et al., 2021; Hong et al., 2022). Owing to their similar morphological characteristics and chemical composition, *Artemisia* species are often mixed or substituted in different regions of China. According to the Chinese flora, *Artemisia princeps* and *A. lancea* are often mixed with *A. argyi.* It is difficult to distinguish dry herbs and raw materials using traditional identification methods. These difficulties have seriously hindered their development as medicinal plants. Some universal DNA barcodes, such as internal transcribed spacer (ITS) and ITS2, have been used to distinguish *Artemisia* species; however, these are inadequate for solving the classification problem because the sequences of closely related species are similar due to the hybridization of *Artemisia* plants (Garcia et al., 2008; Wang et al., 2016; Liu et al., 2017). Therefore, development of an accurate and effective method to identify medicinal *Artemisia* species is urgently needed.

The chloroplast (cp) is a multifunctional organelle with its independent genetic material. The structure of most angiosperm cp genomes is mostly conservative with a typical double-stranded, circular quadripartite structure, which includes a small single copy (SSC) region, large single copy (LSC) region, and two inverted repeat regions (IRa and IRb) (Jansen et al., 2005). With the development of sequencing technology, an increasing number of cp genomes have been published. The cp genome is usually 110–170 kb long, with 110–150 coding genes, which are highly conserved in gene type, gene number, and sequence compared with the mitochondrial or nuclear genome (Green, 2011; Zhu et al., 2016). The evolution rate of the cp genome is relatively moderate (Dong et al., 2013). Due to the lack of recombination, small genome size, and high single cell copy number, the cp genome is widely used in phylogenetic analysis and species identification (Dong et al., 2012; Twyford and Ness, 2017; Dong et al., 2017; Dong et al., 2018; Wu et al., 2018; Mader et al., 2018; Yang et al., 2018; Zhang et al., 2019). The comparison of cp genomes helps discover sequence variations, such as simple sequence repeats (SSRs), and mutation hotspots, and this has led some researchers to propose that the cp genome can be used as a super barcode for species identification (Li et al., 2015). Compared with the traditional relatively short and easily amplified DNA barcode, the cp genome has more abundant mutation site information and stronger species resolution ability, which can more accurately reflect the genetic characteristics of closely related species.

In this study, we used a second-generation sequencing platform to obtain the cp genomes from five *Artemisia* species. We compared their genome structure, codon usage preference, repeat sequences, and mutation hotspots. Finally, we performed a phylogenetic analysis of 29 cp genomes from 19 angiosperms. This study aimed to contribute valuable information toward the construction of the cp genome database of *Artemisia* species, which will aid in their identification.

## Materials and methods

### Sample collection, DNA extraction, and sequencing

Fifteen cp genomes from five *Artemisia* species were used in this study (**Supplementary Table S1**). Fresh leaves of 12 individuals from 5 *Artemisia* species were collected from Hainan, Hubei, and Beijing in China. The cp genomes of two additional individuals were downloaded from NCBI (Accession No.: MZ151340.1 and MW411453.1, *A. lactiflora*) and one cp genome was obtained from our previously published study (Accession No.: ON381734, *A. indica*) (Lan et al., 2022). The genomic DNA of each individual was extracted from fresh leaves using the plant DNA Extraction Kit (QIAGEN, Germany). The quality and concentration of genomic DNAs were evaluated using the Qubit2.0 Fluorometer (Thermo Scientific, USA) and NanoDrop 2000c spectrophotometer (Nanodrop Technologies, Wilmington, DE, USA) to ensure they met the requirements for sequencing. Based on the Illumina Nova Seq sequencing platform, 2 × 150 bp sequencing was performed with a depth of 226–578×. After quality pruning, clean reads were obtained from the original sequencing data for subsequent splicing and annotation.

### Genome assembly and annotation

The NOVOPlasty software (https://github.com/ndierckx/NOVOPlasty) was used to assemble the cp genomes. We compared the clean reads with the scaffold obtained from the assembly, optimized the assembly results according to the paired-end and overlap relationships of the reads, and used the GapCloser software (v1.12, http://soap.genomics.org.cn/soapdenovo.html) to repair the inner hole of the assembly result. Finally, the reference genome was used to correct the starting position of the assembled cp sequence and determine the position and direction of four cp partitions (LSC/IRa/SSC/IRb) for obtaining the final cp genome sequence. The cp genomes were annotated using CpGAVAS (Liu et al., 2012). The genome circle maps were drawn using the online tool OGDRAWH (http://ogdraw.mpimp-golm.mpg.de/) (Lohse et al., 2007). The cp genomes and gene annotation files were uploaded to the NCBI database to obtain GenBank accession numbers.

### Codon usage analysis

The relative synonymous codon usage (RSCU) of the 15 cp genomes from the 5 *Artemisia* species was determined and analyzed using the CodonW1.4.2 software (http://mobyle.pasteur.fr/cgi-bin/portal.py?form=codonw). Heat maps were constructed using the RSCU values. An RSCU value of >1 indicates that the codon is used more frequently, a value equal to 1 indicates that the codon has no usage preference, and a value of <1 indicates that the codon is used less frequently.

### Repeat sequences and simple sequence repeat analysis

Four types of repeat sequences—forward, reverse, complementary, and palindromic—were identified using REPuter with a Hamming distance of 3 and a minimum repeat size of 30 bp (Kurtz et al., 2001). SSRs were detected using MISA with the following parameters: eight repeat units for mononucleotides; four for di- and trinucleotides; and three for tetra-, penta-, and hexanucleotides (Thiel et al., 2003; Lin et al., 2012).

### Nucleotide diversity analysis

The nucleotide diversity of the 15 cp genomes was calculated *via* sliding window analysis using the DnaSP v5.10 software. The window length was set to 600 bp and the step length to 200 bp (Zhang et al., 2021).

### Phylogenetic analysis

The phylogenetic tree was constructed based on the 15 whole cp genomes from 5 *Artemisia* species, another 13 species, and 1 outgroup *Cirsium japonicum*. All genomes, expect the 12 newly sequenced genomes, were downloaded from NCBI. The 29 cp genomes were compared using the MAFFT software (http://mafft.cbrc.jp/alignment/software/). Phylogenetic analysis was performed using the maximum likelihood (ML) method. Using IQtree’s default parameters, ModelFinder automatically filters the best model to build the ML tree (Bootstrap to 1000) (Nguyen et al., 2015).

## Results

### Chloroplast genome sequencing and features of five *Artemisia* species

The cp genome lengths of *A. lancea*, *A. princeps*, *A. lactiflora*, *A. indica*, and *A. argyi* were 151,132 bp, 151,154 bp, 151,178 bp, 151,161 bp, and 151,152 bp, respectively (**Table 1** and **Supplementary Table S1**). These genomes have a typical quadripartite structure, including an LSC (82,870–82,911 bp), SSC (18,338–18,354 bp), and two IR (24,960–24,961 bp) regions (**Table 1**). The average GC content was 37.5%, and the IR regions possessed higher GC content (43.1%) than the LSC (35.5%–35.6%) and SSC (30.9%) regions. In this study, we annotated 132 genes from the 15 cp genomes, of which 7 tRNA genes, 4 rRNA genes, and 7 protein-coding genes were duplicated in the IR region. A total of 114 genes were unique, including 80 protein-coding, 30 tRNA, and 4 rRNA genes.

**Table 1:** Basic cp genome information of five *Artemisia* species.

Characteristics	*A. lancea*	*A. princeps*	*A. lactiflora*	*A. indica*	*A. argyi*
Raw data no.	39,907,620	39,570,956	35,584,122	31,794,994	34,216,492
Chloroplast genome coverage (×)	246	226	325	256	578
Total size (bp)	151,132	151,154	151,178	151,161	151,152
LSC length (bp)	82,870	82,880	82,911	82,901	82,891
IR length (bp)	24,960	24,960	24,960	24,961	24,960
SSC length (bp)	18,342	18,354	18,347	18,338	18,341
Total genes	132	132	132	132	132
Protein coding genes	87	87	87	87	87
tRNA genes	37	37	37	37	37
rRNA genes	8	8	8	8	8
Overall GC content (%)	37.5	37.5	37.5	37.5	37.5
GC content in LSC (%)	35.6	35.5	35.5	35.5	35.6
GC content in IR (%)	43.1	43.1	43.1	43.1	43.1
GC content in SSC (%)	30.9	30.9	30.9	30.9	30.9

### Relative synonymous codons usage

**Figure 1:**
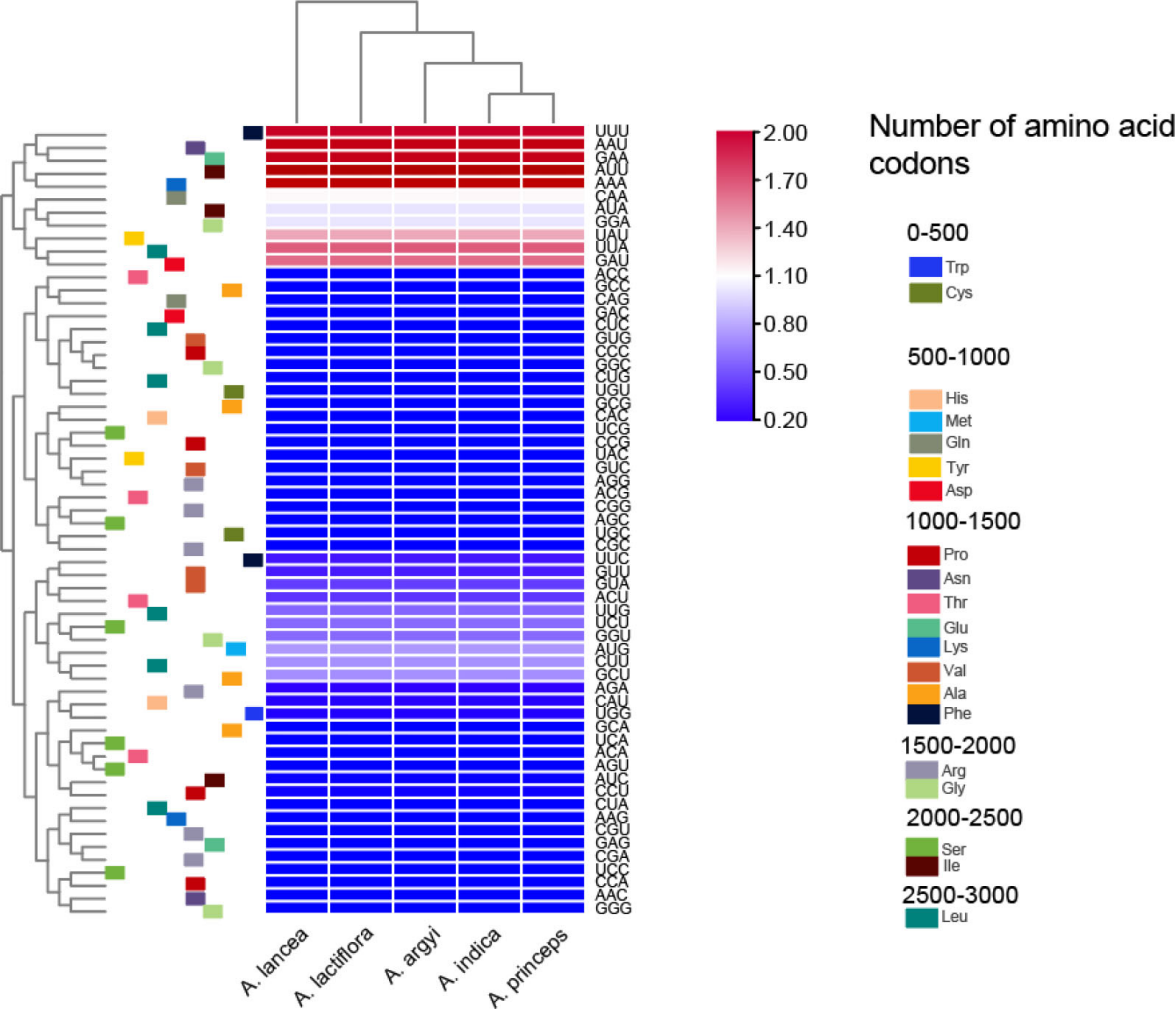
Heat map of the relative synonymous codons usage values of the cp genomes in the five *Artemisia* species.

The cp genomes of the 5 *Artemisia* species contained 64 codons. Of these, 61 codons encoded 20 proteins, and the other 3 were termination codons. The codon AUU had the highest usage frequency (1,078–1,079) with an RSCU value of 1.46–1.47. The RSCU values of the cp genomes in the five species were slightly different. Methionine (Met) and tryptophan (Trp) were encoded by a single codon, with an RSCU value of 1, indicating no preference. All other amino acids were encoded by multiple codons (**Figure 1** and **Supplementary Table S2**). Arg, Leu, and Ser were encoded by six codons; alanine (Ala), glycine (Gly), proline (Pro), threonine (Thr), and valine (Val) by four codons; isoleucine (Ile) by three codons; and the rest were encoded by two codons.

### Repeat and simple sequence repeat analyses

**Figure 2:**
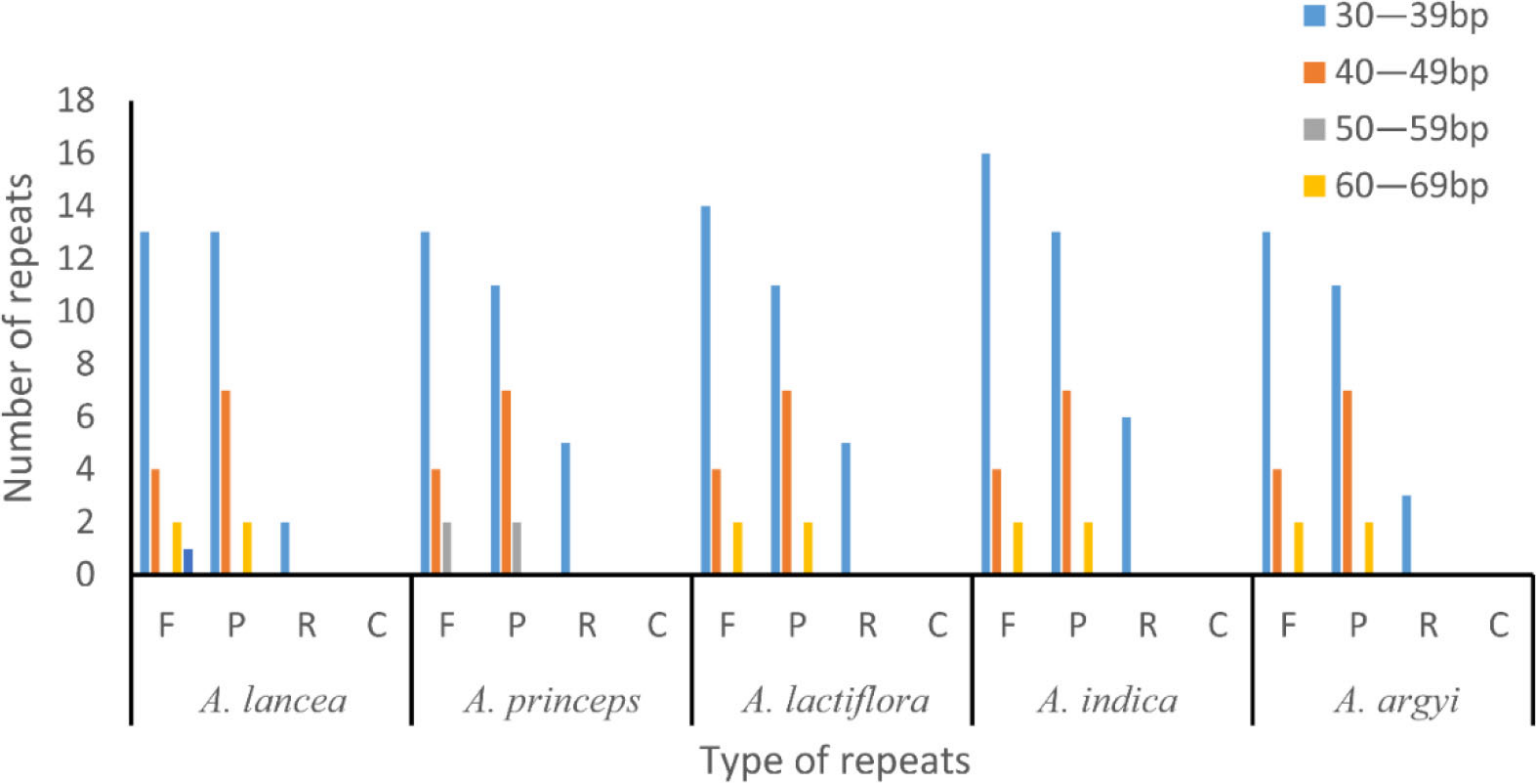
Long repeat sequence analysis of the genomes of five *Artemisia* species. F, forward repeat; P, palindromic repeat; R, reverse repeat; C, complementary repeat.

Some repeats with a length of ≥30 bp are known as long repeats. These are conducive to cp genome rearrangements and increase the genetic diversity of the population. In total, we found 42–50 long repeats in the 15 cp genomes of the 5 *Artemisia* species, including 19–22 forward, 20–22 palindromic, and 3–6 reverse repeats. Most of these repeats, which were 30–39 bp long, were located in the gene spacer and intron regions. This length of repeats was dominant in the *Artemisia* cp genomes, and the longest was the forward repeat. Complementary repeats were not identified in these genomes (**Figure 2**).

**Figure 3:**
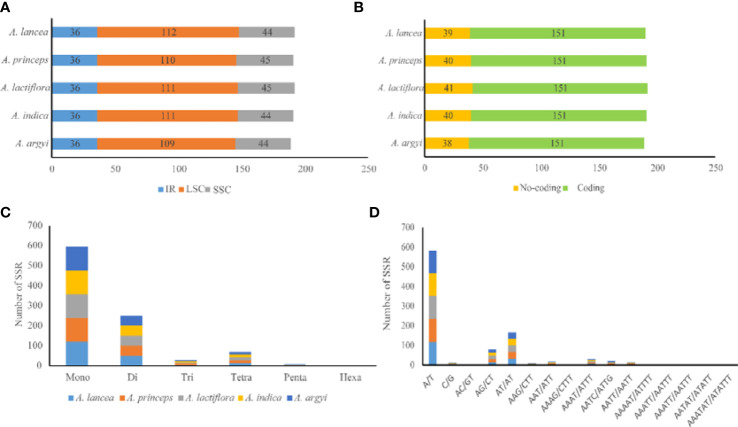
Type and distribution of SSRs in the five *Artemisia* cp genomes. **(A)** Frequency of SSRs in the LSC, SSC, and IR regions. **(B)** SSR distribution between coding and noncoding regions. **(C)** Number of SSR types. **(D)** Number of identified SSR motifs in different repeat class types. SSR, simple sequence repeat; LSC, large single copy region; SSC, small single copy region; IR, inverted repeat region.

SSRs mainly comprise 1–6 types of nucleotide repeats. The cp genome exhibits characteristics of parthenogenesis, and SSRs highly vary within the same species. Therefore, SSR is widely used as a molecular marker in genetic map construction, target gene calibration, and mapping. We observed a total of 189–192 SSRs in the cp genomes of the 5 *Artemisia* species. Of these, 118–121 were mononucleotide SSRs, and most of them were of the A/T type (**Figure 3** and **Supplementary Table S3**). The numbers of di-, tri-, tetra-, penta-, and hexanucleotide SSRs were 50–51, 5–6, 14–15, 1–2, and 0–1, respectively. Using comparative analysis, we found that the five *Artemisia* species had similar SSRs; however, the pentanucleotide repeat AAAAT/ATTTT only existed in *A. argyi* and the hexanucleotide repeat AAATAT/ATATTT only existed in *A. indica*. Overall, most SSRs comprised mono- or dinucleotide repeats. The types of oligonucleotide repeats were rich (**Figure 3**).

### Comparative analysis of the Cp genome

**Figure 4:**
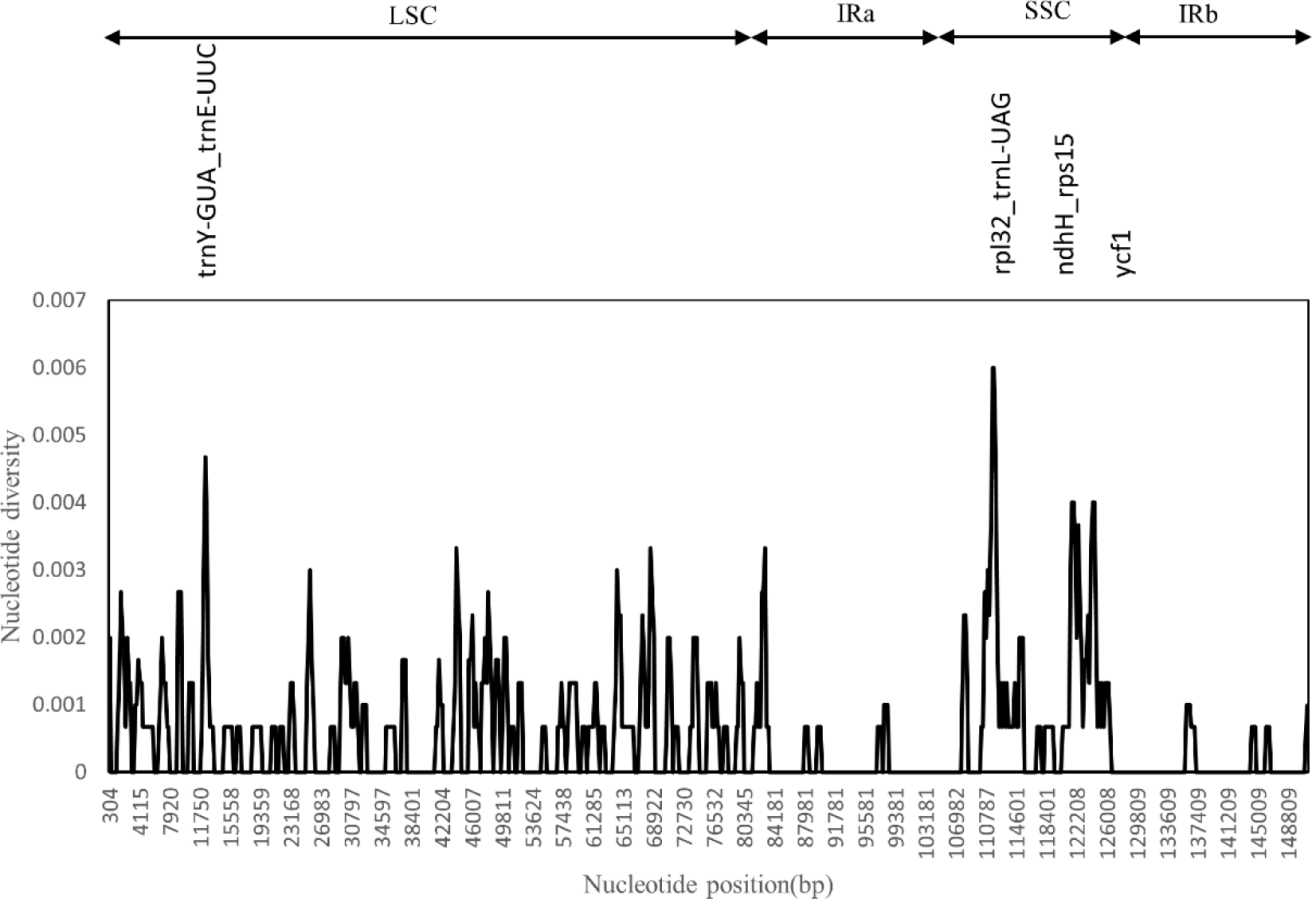
Sliding window test of nucleotide diversity (Pi) in the multiple alignments of the five *Artemisia* cp genomes. Peak regions with a Pi value of >0.004 were labeled with loci tags of the genic or intergenic region names. Pi values were calculated in the 600 bp sliding windows with steps of 200 bp. LSC, large single copy region; IRa, inverted repeat region a; SSC, small single copy region; IRb, inverted repeat region b.

We used the DnaSP software to compare the nucleotide variation values (Pi) between all genes and intergenic regions in the cp genomes of the five *Artemisia* species. The hypervariable regions were detected, and the sequence differences were analyzed. Sliding window analysis revealed that the nucleotide diversity values within 600 bp varied from 0 to 0.006. Four mutational hotspots in the LSC and SSC regions were identified, including *rpl32_trnL-UAG*, *trnY-GUA_trnE-UUC*, *ndhH_rps15*, and *ycf1* (**Figure 4**). These can be used as potential sites for studying population genetics and the identification of *Artemisia* species.

### Phylogenetic analysis

**Figure 5:**
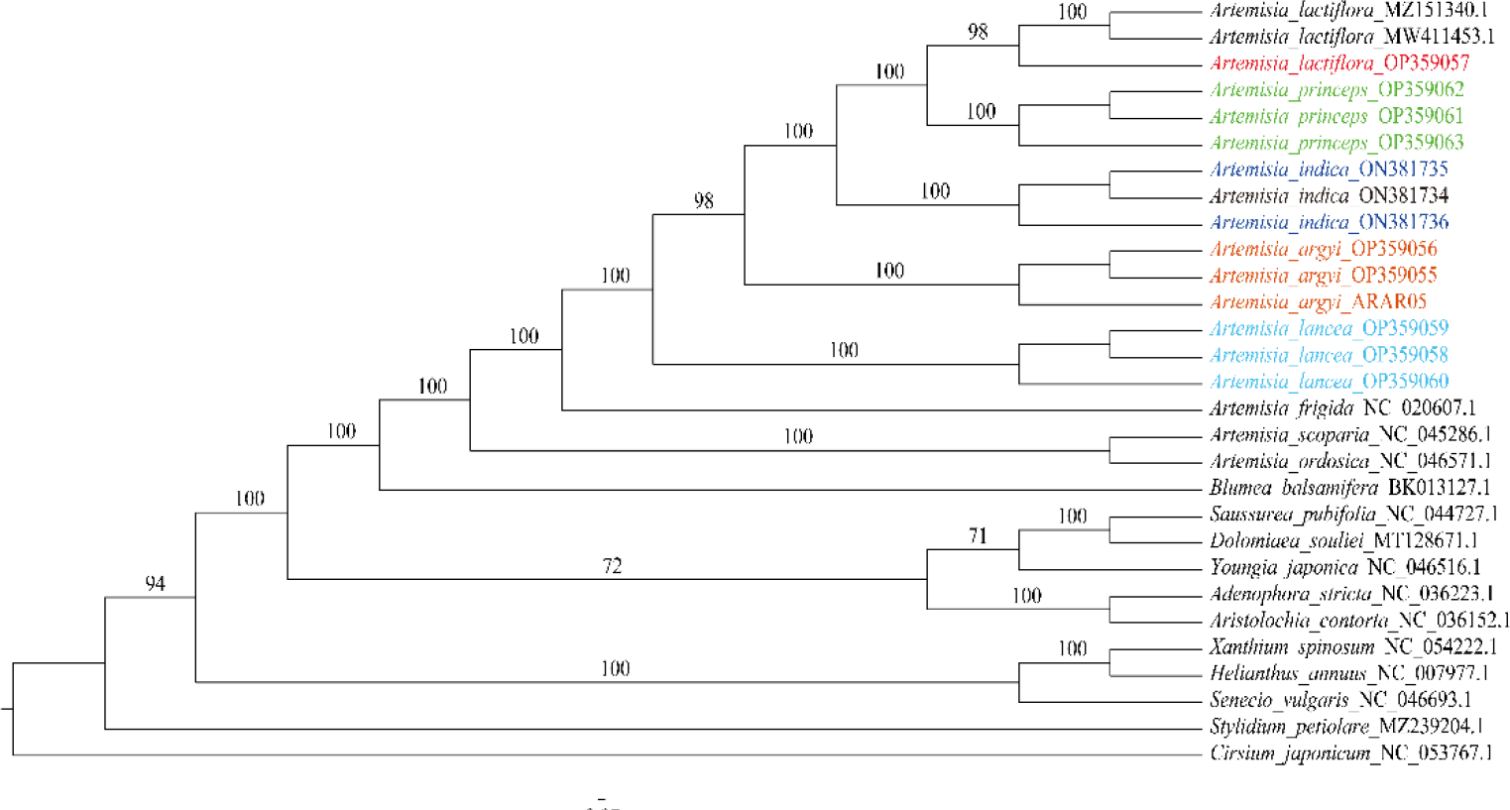
Phylogenetic tree constructed using the ML method based on the 29 cp genomes from 19 species. The numbers above the branches represent the ML bootstrap values. ML, maximum likelihood.

We constructed an ML tree using 29 cp genomes: 15 from the 5 *Artemisia* species used in this study and others from another 13 species and 1 outgroup. We found that all *Artemisia* species clustered together, and different repeat individuals in each species formed a monophyletic branch with a high branch supporting rate, indicating that the cp genome could distinguish the five *Artemisia* species. *A. lactiflora* showed the closest relationship to *A. princeps*, followed by *A. indica* and *A. argyi*, and was distant from *A. lancea* (**Figure 5**). *A. scoparia* and *A. ordosica* were grouped into one branch, revealing a close relationship between them.

## Discussion

In this study, we reported 12 newly sequenced cp genomes from 5 *Artemisia* medicinal species. We found that the genomes were extremely similar, with their size ranging from 151,132–151,178 bp. They belonged to medium-sized cp genomes compared to other Asteraceae species. The cp genomes of five *Artemisia* species contain 114 genes, which was similar to those of *Artemisia annua* (Shen et al., 2017). Like most other *Artemisia* species, ycf1 and rps19 were also detected, but the copy number and location were different, ycf1 spans the IRb/SSC boundary, this was also seen in *A. scoparia* and *A. absinthium* (Chen et al., 2022). The GC content of the IR region was significantly greater than that of the LSC and SSC regions. The AT content was higher than the GC content in all cp genomes. As observed in most plants, we found that the cp genome of *Artemisia* was conservative and no rearrangements were detected in the five species. Multiple codons that encode the same amino acid are known as synonymous codons. Codon usage is unequal, as some synonymous codons are used more frequently than others, a phenomenon known as codon preference. Codon preferences develop in the long-term evolution of plants, and different species have distinct preferences. In this study, we found that the amino acid Leu had the highest proportion of codons in the cp genomes of the five *Artemisia* species.

SSRs widely exist in the cp genome and provide important information regarding population genetics and evolution. Their types, numbers, and distribution vary in each plant. In this study, 57.29%–58.33% of the SSRs were mapped to the LSC region. An SSR-rich region may harbor mutational hotspots (George et al., 2015). The A/T type accounted for the largest proportion of SSRs. The obvious nucleotide bias may be due to the lower number of hydrogen bonds and lower energy consumption of A/T bases (Niu et al., 2017; Kim and Cheon, 2021). Previous studies have reported a higher A/T than G/C content in most plants, which may be due to the large number of A/T-type SSRs (Wang et al., 2021; Wu et al., 2021; Han et al., 2022). In this study, we analyzed the number, location, and composition of SSRs in the cp genomes of five *Artemisia* species and provided a new reference for further research on molecular markers, mutation hotspots, population genetics, and crop breeding.

The mutation hotspot analysis revealed a high degree of similarity among the cp genomes of the five *Artemisia* species, implying that the differentiation of these species was lower than that of other species. The lack of genome information has hindered the classification, identification, and protection of *Artemisia* species. The cp genome sequence provides a basis for the further study on genome evolution and the development of genetic resources. Mutation hotspots are often used for species identification, and these highly variable regions can serve as specific DNA barcodes. In this study, we identified four hypervariable regions—*rpl32_trnL-UAG*, *trnY-GUA_trnE-UUC*, *ndhH_rps15*, and *ycf1*—all of which have the potential to be used as DNA barcodes for subsequent studies on *Artemisia* species.

Phylogenetic analysis is extremely important for clarifying the genetic relationship between species and for protecting, rationally developing, and utilizing plant resources. The cp genome can solve some issues that morphological taxonomy cannot; hence, it has widely been used to explore the phylogenetic relationships between species (Kim Y. K. et al., 2020; Zhao et al., 2021). Due to the low degree of genetic differentiation and similar morphology of *Artemisia* species, obtaining more information on the genetic features of *Artemisia* is expected to improve phylogenetic resolution. In this study, using phylogenetic analysis, we showed that *Artemisia* was a branch of Asteraceae, the five *Artemisia* species are sister groups that can distinguish each other and different repeat individuals in each *Artemisia* species formed a monophyletic branch, indicating that the cp genome can be used as a super barcode to distinguish the five *Artemisia* species. The cp genome provided an effective marker for inferring the phylogenetic relationships between *Artemisia* species.

## Conclusion

In this study, we analyzed 15 cp genomes from 5 *Artemisia* species, including 12 newly sequenced genomes from *A. argyi*, *A. lactiflora*, *A. indica*, *A. princeps*, and *A. lancea*, all of which have been used as medicinal plants for a long time. The cp genomes were similar in structure and gene content and were highly conserved. Four hotspot regions and 189–192 SSR molecular markers were identified, which can serve as potential DNA barcodes for further studies on *Artemisia* species. The phylogenetic analysis showed that the entire cp genome provides distinguishing features to help identify the five *Artemisia* species with high support rates. This study will contribute to the study of population genetics, species identification, and conservation biology of *Artemisia* species.

## Data availability statement

The data presented in the study are deposited in the NCBI repository, accession numbers OP359055–63, and ON381735–36.

## Author contributions

LW and LX designed the research study. ZL and RG performed the research. LW, QY, YS, JL, and YN collected *Artemisia* plant materials. ZL, CL, WW, and XT analyzed the data. ZL and LW wrote and revised the manuscript. All authors contributed to the article and approved the submitted version.

## Funding

This study was supported by the National Natural Science Foundation of China (U1812403-1 and 81903758), the CACMS Innovation Fund (CI2021A05103 and CI2021A04112), the Fundamental Research Funds for the Central public welfare research institutes (ZZ13-YQ-106).

## Acknowledgments

I would like to thank HT, GD, and TW for their valuable guidance during this research.

## Conflict of interest

Authors JL and YN were employed by the company Hubei Aiaitie Health Technology Co., LTD.

The remaining authors declare that the research was conducted in the absence of any commercial or financial relationships that could be construed as a potential conflict of interest.

## Publisher’s note

All claims expressed in this article are solely those of the authors and do not necessarily represent those of their affiliated organizations, or those of the publisher, the editors and the reviewers. Any product that may be evaluated in this article, or claim that may be made by its manufacturer, is not guaranteed or endorsed by the publisher.

## Supplementary material

Supplementary Table 1 The cp genome information of the five *Artemisia* species.

Supplementary Table 2 Relative synonymous codon usage values of the five *Artemisia* species.

Supplementary Table 3 Simple sequence repeat types in the five *Artemisia* species.

## Abbreviations

IR, inverted repeat region; LSC, large single copy region; SSC, small single copy region; ML, maximum likelihood; rRNA, ribosomal RNA; SSR, simple sequence repeats; tRNA, transfer RNA; RSCU, relative synonymous codon usage.

## References

[B1] Chen C. Miao Y. Luo D. Li J. Wang Z. Luo M.. (2022). Sequence characteristics and phylogenetic analysis of the *Artemisia argyi* chloroplast genome. Front. Plant Sci. 13, 906725. doi: 10.3389/fpls.2022.906725 35795352 PMC9252292

[B2] Chen J. Xu X. Lin L. Guo D. Gui W. Lin Y.. (2021). Research progress on pharmacological effects of artemisia argyi. J. Pharm. Res. 40, 5. doi: 10.13506/j.cnki.jpr.2021.12.009

[B3] Dong W. Liu J. Yu J. Wang L. Zhou S. (2012). Highly variable chloroplast markers for evaluating plant phylogeny at low taxonomic levels and for DNA barcoding. PloS One 7, e35071. doi: 10.1371/journal.pone.0035071 22511980 PMC3325284

[B4] Dong W. Xu C. Cheng T. Lin K. Zhou S. (2013). Sequencing angiosperm plastid genomes made easy: A complete set of universal primers and a case study on the phylogeny of saxifragales. Genome Biol. Evol. 5, 989–997. doi: 10.1093/gbe/evt063 23595020 PMC3673619

[B5] Dong W. Xu C. Li W. Xie X. Lu Y. Liu Y.. (2017). Phylogenetic resolution in juglans based on complete chloroplast genomes and nuclear DNA sequences. Front. Plant Sci. 8, 1148. doi: 10.3389/fpls.2017.01148 28713409 PMC5492656

[B6] Dong W. Xu C. Wu P. Cheng T. Yu J. Zhou S.. (2018). Resolving the systematic positions of enigmatic taxa: Manipulating the chloroplast genome data of saxifragales. Mol. Phylogenet. Evol. 126, 321–330. doi: 10.1016/j.ympev.2018.04.033 29702217

[B7] Garcia S. Canela M. A. Garnatje T. Mcarthur E. D. Pellicer J. Sanderson S. C.. (2008). Evolutionary and ecological implications of genome size in the north American endemic sagebrushes and allies (Artemisia, asteraceae). Biol. J. Linn. Soc. 94, 631–649. doi: 10.1111/j.1095-8312.2008.01001.x

[B8] George B. Bhatt B. S. Awasthi M. George B. Singh A. K. (2015). Comparative analysis of microsatellites in chloroplast genomes of lower and higher plants. Curr. Genet. 61, 665–677. doi: 10.1007/s00294-015-0495-9 25999216

[B9] Green B. R. (2011). Chloroplast genomes of photosynthetic eukaryotes. Plant J. 66, 34–44. doi: 10.1111/j.1365-313X.2011.04541.x 21443621

[B10] Han H. Qiu R. Liu Y. Zhou X. Gao C. Pang Y.. (2022). Analysis of chloroplast genomes provides insights into the evolution of agropyron. Front. Genet. 13, 832809. doi: 10.3389/fgene.2022.832809 35145553 PMC8821885

[B11] Hong F. Zhao M. Xue L. L. Ma X. Liu L. Cai X. Y.. (2022). The ethanolic extract of artemisia anomala exerts anti-inflammatory effects *via* inhibition of NLRP3 inflammasome. Phytomedicine 102, 154163. doi: 10.1016/j.phymed.2022.154163 35597027

[B12] Hsueh T. P. Lin W. L. Dalley J. W. Tsai T. H. (2021). The pharmacological effects and pharmacokinetics of active compounds of artemisia capillaris. Biomedicines 10, 1412. doi: 10.3390/biomedicines9101412 PMC8533588 34680529

[B13] Jansen R. K. Raubeson L. A. Boore J. L. Depamphilis C. W. Chumley T. W. Haberle R. C.. (2005). Methods for obtaining and analyzing whole chloroplast genome sequences. Methods Enzymol. 395, 348–384. doi: 10.1016/S0076-6879(05)95020-9 15865976

[B14] Kim K. A. Cheon K. S. (2021). Complete chloroplast genome sequence of adenophora racemosa *(Campanulaceae)*: Comparative analysis with congeneric species. PloS One 16, e0248788. doi: 10.1371/journal.pone.0248788 33735287 PMC7971521

[B15] Kim Y. K. Jo S. Cheon S. H. Joo M. J. Kim K. J. Hong J. R.. (2020). Plastome evolution and phylogeny of orchidaceae, with 24 new sequences. Front. Plant Sci. 11, 22. doi: 10.3389/fpls.2020.00022 32153600 PMC7047749

[B16] Kim G. B. Lim C. E. Kim J. S. Kim K. Lee J. H. Yu H. J.. (2020). Comparative chloroplast genome analysis of artemisia *(Asteraceae)* in East Asia: Insights into evolutionary divergence and phylogenomic implications. BMC Genomics 21, 415. doi: 10.1186/s12864-020-06812-7 32571207 PMC7310033

[B17] Kurtz S. Choudhuri J. V. Ohlebusch E. Schleiermacher C. Stoye J. Giegerich R. (2001). REPuter: The manifold applications of repeat analysis on a genomic scale. Nucleic Acids Res. 29, 4633–4642. doi: 10.1093/nar/29.22.4633 11713313 PMC92531

[B18] Lan Z. Tian X. Shi Y. Gao R. Yin Q. Xiang L.. (2022). Chloroplast genome structure characteristics and phylogenetic analysis of artemisia indica. China J. Chin. Materia. Med., 47, 224–231. doi: 10.19540/j.cnki.cjcmm.20220713.101 36471930

[B19] Lin C. P. Wu C. S. Huang Y. Y. Chaw S. M. (2012). The complete chloroplast genome of ginkgo biloba reveals the mechanism of inverted repeat contraction. Genome Biol. Evol. 4, 374–381. doi: 10.1093/gbe/evs021 22403032 PMC3318433

[B20] Liu G. Ning H. Ayidaerhan N. Aisa H. A. (2017). Evaluation of DNA barcode candidates for the discrimination of artemisia l. Mitochondrial. DNA Part A. 28, 956–964. doi: 10.1080/24701394.2016.1219729 27607516

[B21] Liu C. Shi L. Zhu Y. Chen H. Zhang J. Lin X.. (2012). CpGAVAS, an integrated web server for the annotation, visualization, analysis, and GenBank submission of completely sequenced chloroplast genome sequences. BMC Genomics 13, 715. doi: 10.1186/1471-2164-13-715 23256920 PMC3543216

[B22] Li X. Yang Y. Henry R. J. Rossetto M. Wang Y. Chen S. (2015). Plant DNA barcoding: from gene to genome. Biol. Rev. Camb. Philos. Soc 90, 157–166. doi: 10.1111/brv.12104 24666563

[B23] Lohse M. Drechsel O. Bock R. (2007). OrganellarGenomeDRAW (OGDRAW): a tool for the easy generation of high-quality custom graphical maps of plastid and mitochondrial genomes. Curr. Genet. 52, 267–274. doi: 10.1007/s00294-007-0161-y 17957369

[B24] Mader M. Pakull B. Blanc-Jolivet C. Paulini-Drewes M. Bouda Z. H. Degen B.. (2018). Complete chloroplast genome sequences of four meliaceae species and comparative analyses. Int. J. Mol. Sci. 19, 701. doi: 10.3390/ijms19030701 29494509 PMC5877562

[B25] Nguyen L. T. Schmidt H. A. von Haeseler A. Minh B. Q. (2015). IQ-TREE: A fast and effective stochastic algorithm for estimating maximum-likelihood phylogenies. Mol. Biol. Evol. 32, 268–274. doi: 10.1093/molbev/msu300 25371430 PMC4271533

[B26] Niu Z. Xue Q. Wang H. Xie X. Zhu S. Liu W.. (2017). Mutational biases and GC-biased gene conversion affect GC content in the plastomes of dendrobium genus. Int. J. Mol. Sci. 18, 2307. doi: 10.3390/ijms18112307 29099062 PMC5713276

[B27] Riggins C. (2008). Molecular phylogenetic and biogeographic study of the genus artemisia (Asteraceae), with an emphasis on section absinthium [D] (USA:University of Illinois at Urbana-Champaign).

[B28] Shen X. Wu M. Liao B. Liu Z. Bai R. Xiao S.. (2017). Complete chloroplast fenome sequence and phylogenetic analysis of the medicinal plant artemisia annua. Molecules 22, 1330. doi: 10.3390/molecules22081330 28800082 PMC6152406

[B29] Thiel T. Michalek W. Varshney R. K. Graner A. (2003). Exploiting EST databases for the development and characterization of gene-derived SSR-markers in barley (Hordeum vulgare l.). Theor. Appl. Genet. 106, 411–422. doi: 10.1007/s00122-002-1031-0 12589540

[B30] Twyford A. D. Ness R. W. (2017). Strategies for complete plastid genome sequencing. Mol. Ecol. Resour. 17, 858–868. doi: 10.1111/1755-0998.12626 27790830 PMC6849563

[B31] Wang Y. Wang S. Liu Y. Yuan Q. Sun J. Guo L. (2021). Chloroplast genome variation and phylogenetic relationships of atractylodes species. BMC Genomics 22, 103. doi: 10.1186/s12864-021-07394-8 33541261 PMC7863269

[B32] Wang X. Zheng S. Liu Y. Han J. (2016). ITS2, a better DNA barcode than ITS in identification of species in artemisia l. Chin. Herbal. Medicines 8, 352–358. doi: 10.1016/S1674-6384(16)60062-X

[B33] Watson L. E. Bates P. L. Evans T. M. Unwin M. M. Estes J. R. (2002). Molecular phylogeny of subtribe artemisiinae (Asteraceae), including artemisia and its allied and segregate genera. BMC Evol. Biol. 2, 17. doi: 10.1186/1471-2148-2-17 12350234 PMC130036

[B34] Wu M. L. Li Q. Xu J. Li X. W. (2018). Complete chloroplast genome of the medicinal plant amomum compactum: gene organization, comparative analysis, and phylogenetic relationships within zingiberales. Chin. Med. 13, 10. doi: 10.1186/s13020-018-0164-2 29449878 PMC5811967

[B35] Wu L. Wu M. Cui N. Xiang L. Li Y. Li X.. (2021). Plant super-barcode: a case study on genome-based identification for closely related species of fritillaria. Chin. Med. 16, 52. doi: 10.1186/s13020-021-00460-z 34225754 PMC8256587

[B36] Yang Z. Zhao T. Ma Q. Liang L. Wang G. (2018). Comparative genomics and phylogenetic analysis revealed the chloroplast genome variation and interspecific relationships of corylus (Betulaceae) species. Front. Plant Sci. 9, 927. doi: 10.3389/fpls.2018.00927 30038632 PMC6046460

[B37] Zhang L. Wang S. Su C. Harris A. J. Zhao L. Su N.. (2021). Comparative chloroplast genomics and phylogenetic analysis of zygophyllum (Zygophyllaceae) of China. Front. Plant Sci. 12, 723622. doi: 10.3389/fpls.2021.723622 34630471 PMC8500179

[B38] Zhang T. Xing Y. Xu L. Bao G. Zhan Z. Yang Y.. (2019). Comparative analysis of the complete chloroplast genome sequences of six species of pulsatilla miller, ranunculaceae. Chin. Med. 14, 53. doi: 10.1186/s13020-019-0274-5 31798674 PMC6883693

[B39] Zhao F. Chen Y. P. Salmaki Y. Drew B. T. Wilson T. C. Scheen A. C.. (2021). An updated tribal classification of lamiaceae based on plastome phylogenomics. BMC Biol. 19, 2. doi: 10.1186/s12915-020-00931-z 33419433 PMC7796571

[B40] Zhu A. Guo W. Gupta S. Fan W. Mower J. P. (2016). Evolutionary dynamics of the plastid inverted repeat: The effects of expansion, contraction, and loss on substitution rates. New Phytol. 209, 1747–1756. doi: 10.1111/nph.13743 26574731

