# Supplementary material for: Comparative and phylogenetic analysis of complete chloroplast genomes from five Artemisia species
Source: Front Plant Sci. 2022 Nov 21;13:1049209. doi: 10.3389/fpls.2022.1049209 (PMC9720176; doi:10.3389/fpls.2022.1049209)
Supplement: Supplementary Table 1 — The cp genome information of the five Artemisia species. [file DataSheet_1.docx]

*Supplementary Material*

**Supplementary Table S1**. The cp genome information of the five *Artemisia* species.

| No. | Species | Sequence Length | Voucher No.^a^ | Accession  No. | Sample  from |
| --- | --- | --- | --- | --- | --- |
| 1 | *A. lactiflora* | 151159 |  | MZ151340.1^b^ |  |
| 2 | *A. lactiflora* | 151159 |  | MW411453.1^b^ |  |
| 3 | *A. lactiflora* | 151178 | ARLA01 | OP359057 | Hainan |
| 4 | *A. princeps* | 151154 | PRPS01 | OP359061 | Beijing |
| 5 | *A. princeps* | 151154 | PRPS02 | OP359062 | Beijing |
| 6 | *A. princeps* | 151154 | PRPS03 | OP359063 | Beijing |
| 7 | *A. indica* | 151161 |  | ON381734^c^ |  |
| 8 | *A. indica* | 151161 | ARIN02 | ON381735 | Hainan |
| 9 | *A. indica* | 151161 | ARIN03 | ON381736 | Hainan |
| 10 | *A. argyi* | 151152 | ARAR01 | OP359055 | Hainan |
| 11 | *A. argyi* | 151152 | ARAR02 | OP359056 | Hubei |
| 12 | *A. argyi* | 151152 | ARAR05^d^ | - | Hubei |
| 13 | *A. lancea* | 151132 | LANE01 | OP359058 | Beijing |
| 14 | *A. lancea* | 151132 | LANE02 | OP359059 | Beijing |
| 15 | *A. lancea* | 151132 | LANE03 | OP359060 | Beijing |

^a^ new samples in this study

^b^ cp genomes downloaded from GenBank

^c^ cp genome obtained from our previously published study

^d^ not uploaded to NCBI

**Supplementary Table S2.** Relative synonymous codon usage values of the five *Artemisia* species.

| amino acid | codon | *A. lancea* | *A. princeps* | *A. lactiflora* | *A. indica* | *A. argyi* |
| --- | --- | --- | --- | --- | --- | --- |
| Phe | UUU(F) | 1.32 | 1.32 | 1.32 | 1.32 | 1.32 |
|  | UUC(F) | 0.68 | 0.68 | 0.68 | 0.68 | 0.68 |
| Leu | UUA(L) | 1.86 | 1.86 | 1.86 | 1.86 | 1.87 |
|  | UUG(L) | 1.23 | 1.23 | 1.23 | 1.23 | 1.23 |
|  | CUU(L) | 1.32 | 1.32 | 1.32 | 1.32 | 1.32 |
|  | CUC(L) | 0.4 | 0.41 | 0.4 | 0.4 | 0.4 |
|  | CUA(L) | 0.77 | 0.77 | 0.77 | 0.77 | 0.77 |
|  | CUG(L) | 0.41 | 0.41 | 0.41 | 0.41 | 0.41 |
| Ile | AUU(I) | 1.46 | 1.47 | 1.46 | 1.46 | 1.47 |
|  | AUC(I) | 0.59 | 0.59 | 0.59 | 0.59 | 0.59 |
|  | AUA(I) | 0.95 | 0.95 | 0.95 | 0.95 | 0.95 |
| Met | AUG(M) | 1 | 1 | 1 | 1 | 1 |
| Val | GUU(V) | 1.44 | 1.44 | 1.44 | 1.44 | 1.44 |
|  | GUC(V) | 0.49 | 0.48 | 0.49 | 0.49 | 0.49 |
|  | GUA(V) | 1.54 | 1.54 | 1.54 | 1.54 | 1.54 |
|  | GUG(V) | 0.53 | 0.53 | 0.53 | 0.53 | 0.53 |
| Ser | UCU(S) | 1.73 | 1.74 | 1.74 | 1.74 | 1.74 |
|  | UCC(S) | 0.97 | 0.96 | 0.96 | 0.96 | 0.96 |
|  | UCA(S) | 1.23 | 1.23 | 1.23 | 1.23 | 1.23 |
|  | UCG(S) | 0.49 | 0.49 | 0.49 | 0.49 | 0.49 |
|  | AGU(S) | 1.22 | 1.23 | 1.23 | 1.23 | 1.23 |
|  | AGC(S) | 0.35 | 0.35 | 0.35 | 0.36 | 0.35 |
| Pro | CCU(P) | 1.57 | 1.57 | 1.57 | 1.57 | 1.57 |
|  | CCC(P) | 0.68 | 0.68 | 0.68 | 0.68 | 0.67 |
|  | CCA(P) | 1.17 | 1.17 | 1.17 | 1.17 | 1.18 |
|  | CCG(P) | 0.58 | 0.57 | 0.57 | 0.57 | 0.57 |
| Thr | ACU(T) | 1.62 | 1.63 | 1.63 | 1.63 | 1.62 |
|  | ACC(T) | 0.74 | 0.74 | 0.74 | 0.73 | 0.74 |
|  | ACA(T) | 1.25 | 1.26 | 1.26 | 1.26 | 1.25 |
|  | ACG(T) | 0.38 | 0.38 | 0.38 | 0.38 | 0.38 |
| Ala | GCU(A) | 1.75 | 1.75 | 1.75 | 1.75 | 1.75 |
|  | GCC(A) | 0.65 | 0.65 | 0.65 | 0.65 | 0.65 |
|  | GCA(A) | 1.17 | 1.17 | 1.17 | 1.17 | 1.17 |
|  | GCG(A) | 0.43 | 0.44 | 0.44 | 0.44 | 0.44 |
| Tyr | UAU(Y) | 1.64 | 1.64 | 1.64 | 1.64 | 1.64 |
|  | UAC(Y) | 0.36 | 0.36 | 0.36 | 0.36 | 0.36 |
| His | CAU(H) | 1.52 | 1.52 | 1.52 | 1.52 | 1.52 |
|  | CAC(H) | 0.48 | 0.48 | 0.48 | 0.48 | 0.48 |
| Gln | CAA(Q) | 1.52 | 1.52 | 1.52 | 1.52 | 1.52 |
|  | CAG(Q) | 0.48 | 0.48 | 0.48 | 0.48 | 0.48 |
| Asn | AAU(N) | 1.55 | 1.55 | 1.55 | 1.55 | 1.55 |
|  | AAC(N) | 0.45 | 0.45 | 0.45 | 0.45 | 0.45 |
| Lys | AAA(K) | 1.47 | 1.47 | 1.47 | 1.47 | 1.47 |
|  | AAG(K) | 0.53 | 0.53 | 0.53 | 0.53 | 0.53 |
| Asp | GAU(D) | 1.6 | 1.6 | 1.61 | 1.6 | 1.6 |
|  | GAC(D) | 0.4 | 0.4 | 0.39 | 0.4 | 0.4 |
| Glu | GAA(E) | 1.49 | 1.49 | 1.49 | 1.49 | 1.49 |
|  | GAG(E) | 0.51 | 0.51 | 0.51 | 0.51 | 0.51 |
| Cys | UGU(C) | 1.4 | 1.4 | 1.4 | 1.4 | 1.4 |
|  | UGC(C) | 0.6 | 0.6 | 0.6 | 0.6 | 0.6 |
| Trp | UGG(W) | 1 | 1 | 1 | 1 | 1 |
| Arg | CGU(R) | 1.33 | 1.34 | 1.34 | 1.34 | 1.34 |
|  | CGC(R) | 0.4 | 0.4 | 0.4 | 0.4 | 0.4 |
|  | CGA(R) | 1.32 | 1.31 | 1.31 | 1.3 | 1.31 |
|  | CGG(R) | 0.46 | 0.46 | 0.46 | 0.47 | 0.47 |
|  | AGA(R) | 1.82 | 1.82 | 1.82 | 1.83 | 1.83 |
|  | AGG(R) | 0.66 | 0.66 | 0.66 | 0.66 | 0.66 |
| Gly | GGU(G) | 1.31 | 1.31 | 1.31 | 1.31 | 1.31 |
|  | GGC(G) | 0.42 | 0.42 | 0.43 | 0.42 | 0.43 |
|  | GGA(G) | 1.58 | 1.58 | 1.57 | 1.58 | 1.57 |
|  |  |  |  |  |  |  |
|  | GGG(G) | 0.69 | 0.69 | 0.69 | 0.69 | 0.69 |
| Stop | UAA(*) | 1.76 | 1.76 | 1.76 | 1.76 | 1.76 |
|  | UAG(*) | 0.72 | 0.72 | 0.72 | 0.72 | 0.72 |
|  | UGA(*) | 0.52 | 0.52 | 0.52 | 0.52 | 0.52 |

**Supplementary Table S3**. Simple sequence repeat types in the five *Artemisia* species.

| SSR Type | *A. lancea* | *A. princeps* | *A. lactiflora* | *A. indica* | *A. argyi* |
| --- | --- | --- | --- | --- | --- |
| A/T | 118 | 117 | 117 | 116 | 116 |
| C/G | 3 | 2 | 2 | 2 | 2 |
| AC/GT | 1 | 1 | 1 | 1 | 1 |
| AG/CT | 16 | 16 | 16 | 16 | 16 |
| AT/AT | 34 | 33 | 33 | 33 | 33 |
| AAG/CTT | 2 | 2 | 2 | 2 | 2 |
| AAT/ATT | 3 | 4 | 4 | 4 | 3 |
| AAAG/CTTT | 1 | 1 | 1 | 1 | 1 |
| AAAT/ATTT | 6 | 6 | 7 | 6 | 6 |
| AATC/ATTG | 4 | 4 | 4 | 4 | 4 |
| AATT/AATT | 3 | 3 | 3 | 3 | 3 |
| AAAAT/ATTTT | 0 | 0 | 0 | 0 | 1 |
| AAATT/AATTT | 1 | 1 | 0 | 0 | 1 |
| AAATT/AATTT | 0 | 0 | 1 | 1 | 0 |
| AATAT/ATATT | 0 | 1 | 1 | 1 | 0 |
| AAATAT/ATATTT | 0 | 0 | 0 | 1 | 0 |
